# Commentary on Borodovsky *et al*.: Enhancing research on THC quantification—Consumer awareness through accurate labelling

**DOI:** 10.1111/add.16758

**Published:** 2025-01-11

**Authors:** Rachel Lees Thorne, Tom P. Freeman

**Affiliations:** ^1^ Addiction and Mental Health Group, Department of Psychology University of Bath Bath UK

**Keywords:** cannabis use disorder, measurement, product labelling, standard units, THC, THC cannabis potency

*Recent advances in the quantification of Δ‐9‐tetrahydrocannabinol (THC) have the potential to increase our understanding of the effects of cannabis use on health. Policies and regulation supporting accurate and accessible THC content labelling will advance scientific progress and give consumers better control over their use*.

Accurately quantifying use of a drug is vital for assessing its impact on public health. Within the field of cannabis use, quantification has raised major challenges because of the wide range of cannabis products, methods of administration, and distinct policy contexts globally. Recent advances in quantification have focused on THC (Δ‐9‐tetrahydrocannabinol, the main psychoactive component of cannabis). The amount of THC consumed has been linked to negative cannabis‐related consequences, including cannabis use disorder (CUD) and mental ill‐health [1, 2, 3]. By focusing on measurement of THC (rather than widely used measures such as days per week of use), Borodovsky and colleagues [4] provide important advances in estimating the direct relationship between quantity of drug used and health outcomes to inform lower risk guidelines.

Borodovsky and colleagues [4] found that higher daily THC consumption was associated with a greater number of CUD symptoms and higher odds for meeting clinical thresholds for CUD in a large sample of United States (US) respondents. This represents an important step in our ability to estimate dose‐related risk of a key public health outcome that occurs in approximately one in five people who use cannabis [5]. To calculate THC consumption in their comprehensive online survey, participants reported their daily quantity of cannabis use over the past week across a range of product types and estimated the potency of the cannabis that they had used.

Both quantity and potency of cannabis are needed to estimate THC consumption, and both have been linked to important health outcomes [1, 6, 7]. Overlooking these factors in previous research may have affected our understanding of the associations between cannabis and health outcomes such as CUD. However, estimating quantity and potency is not straightforward and requires more in depth questioning than estimating frequency alone. Several assessments, including Borodovsky and colleagues' [4] work, have set out to comprehensively assess THC consumption, however, several factors may influence the accuracy of estimation of these data.

Across the United States, there is a complex picture of differing legal frameworks surrounding the availability and sale of medicinal and/or recreational cannabis. Outside of the United States, most people who use cannabis do so in a setting in which cannabis is not legally sold or purchased. In legal markets, accurate information about cannabis products including quantity and potency can be included on labelling and packaging. In reality, this is not always the case, as cannabis products are not always correctly labelled [8]. Moreover, current labelling may not be accessible enough for consumers to interpret and retain this information. Consequently, many people who use cannabis report that they are not aware of the THC quantity in the products they consume, even in legal markets [9, 10]. Moreover, where cannabis is purchased illegally, consumers may lack accurate information on the cannabis they are purchasing. Researchers often need to rely on participants' recall of this information to estimate their THC consumption and, therefore, this may introduce bias to our estimates. However, recent research indicates that THC consumption can be estimated by collecting data on product type and its average THC concentration, together with amount and frequency of use, with strong validity according to urinary THC concentrations [11]. Nevertheless, we need to understand the influence of contextual factors on participant accuracy of estimation of their THC consumption, as well as encourage better labelling of cannabis products to improve consumers' knowledge.

Furthermore, accurate labelling of products will enable individuals who use cannabis to respond to knowledge generated by research and to any future guidelines for safer use based on THC, as Borodovsky and colleagues [4] recommend. One approach could be through labelling packaging with standard THC units [12] in the same way that alcohol products are labelled with alcohol units in many jurisdictions around the world. To maximise impact on public health, manufacturers and relevant legal bodies should incorporate such measures on accessible labelling for cannabis products, so that those who would like to monitor their use are able to do so accurately. For example, Health Canada recently sought public guidance on simplifying THC labelling on products, and the legislative review of the Cannabis Act recommended that a ‘standard dose’ or ‘unit dose’ should be prioritized and accompanied by regulatory amendments to require it as an element on cannabis product labels [13]. If successfully implemented, such regulation could enhance communication and consumer knowledge about THC.

Increasing the accuracy and accessibility of information available around quantity and potency of cannabis products has the potential to both improve the accuracy of our research on CUD as well as to enable consumers to make more informed choices to reduce their risk.

## DECLARATION OF INTERESTS

None.

## Data Availability

Data sharing is not applicable to this article as no new data were created or analyzed for this commentary.
